# The pain and mental health comorbidity

**DOI:** 10.1017/S204579602400057X

**Published:** 2024-10-03

**Authors:** Ketan Bhatt, Angela Cano Palomares, Liisa Jutila, Iben Rohde, Patrice Forget, The Societal Impact of Pain Platform (SIP)

**Affiliations:** 1 School of Medicine, Medical Sciences and Nutrition, University of Aberdeen, Aberdeen, UK; 2 European Pain Federation EFIC, Societal Impact of Pain Platform (SIP), Brussels, Belgium; 3 Pain Alliance Europe PAE, Brussels, Belgium; 4 Institute of Applied Health Sciences, Epidemiology Group, School of Medicine, Medical Sciences and Nutrition, University of Aberdeen, Aberdeen, UK; 5 Department of Anaesthesia, NHS Grampian, Aberdeen, UK

Pain has had a perennial association with physical ailments, health disorders and injuries since time immemorial. Any form of severe physiological stress or disease triggers episodes of pain which can impact mental health and drastically affect the quality of life. An estimated 20% of the European adult population experiences chronic pain at some point in their life (Breivik *et al.*, 2006). That is a staggering 150 million people with women at an increased risk of suffering from chronic pain than men (Bartley and Fillingim, 2013). As per an OECD report, 1 out of 6 people in the EU are affected by mental health (Ciucci, 2020) amounting to an estimated economic impact of 4% of GDP or EUR 600 billion (D’Acci, 2020). Post COVID-19 pandemic, the incidence is likely to have seen a dramatic rise. The European mental health strategy (EMHS) has therefore been a welcome and timely initiative to advance mental healthcare across the EU.

We appreciate the views of Fioritti and colleagues regarding the importance of focusing on the relationship between mental health and work, both from a preventative aspect and reintegration of those who are at risk of exclusion (Fioritti *et al.*, 2024). We fully recognize the value of social cohesion and its impact on economic growth. However, we would like to complement this by specifically highlighting the priority attention that needs to be given to the double disability burden of mental health and chronic pain.

It is commendable to see the cross-sectoral promotion and integration of mental health across several policy domains in the EMHS. The strategy acknowledges the influence of biological and psychological factors underlying mental health and advocates for factoring-in comorbidities and disabilities in designing effective treatment strategies. However, there is no explicit acknowledgement of the role of comorbidities and the bidirectional relationship between mental health and pain. Evidence suggests a close association between chronic pain and poor mental health states (Bondesson *et al.*, 2018) with several social disadvantages as common denominators. These include poverty, unemployment, low socioeconomic status, lack of access to healthcare services, etc (Kohrt *et al.*, 2018). It has been reported that the risk of chronic pain prevalence is twice as high in people with bipolar disorder compared to the general population (Nicholl *et al.*, 2014). This can place people at a heightened risk of social and workplace exclusion.

Adverse mental health is a frequent concomitant of chronic pain leading to unfavourable immediate and long-term impacts on quality of life and survival. Despite the shared biopsychosocial experiences, pain is not normally assessed and addressed in mental health patients (Brennan *et al.*, 2019). Moreover, undiagnosed and underrecognized mental health conditions in people with chronic pain often go untreated. Mental health and pain are mutually reinforcing. Treating them in isolation and independent of each other would result in unsuccessful treatment outcomes such as continued reduction in quality of life, mobility and social participation. Pain directly interferes with mental health treatments by negatively impacting the overall outcome (Roughan *et al.*, 2021). A multidisciplinary approach to pain management is thus an essential pre-requisite for effective treatment strategies and improved therapy outcomes.

Therefore, the critical need to standardize assessment of pain and recognizing its causal relationship with mental health conditions cannot be overemphasized. This would make it easier to identify people with mental health conditions, living with pain, for prevention and early intervention. Early access to integrated care services involving professionals from multiple disciplines such as psychologists and physiotherapists can then deliver biopsychosocial interventions in the management of chronic pain. These include cognitive behavioural therapies, physical activity programs, relaxation activities, etc. Care givers can augment pain mitigating behaviours by engaging patients with exercise, body awareness programs and emotional regulation activities. However, the stigma attached to mental health and pain creates barriers for recognition and access to care.

Pharmacotherapeutic analgesics such as opioids carry a serious risk of physical dependence and, separately, may cause problems in people who have or are at risk of substance use disorders. Over the last two decades, Europe has witnessed a rise in mortality as a result of opioid prescription and use although the observed trends encompass variability among different countries (Forget and Hauser, 2023). Chronic pain has been found to be a significant problem in half of the people with opioid use disorders. This warrants a serious revisit of quality and delivery of biopsychosocial pain interventions in Europe.

While EMHS recognizes the value of inclusive workplaces and stresses on reintegration, the strategy does not clearly demonstrate its commitment to protect the rights of individuals, with mental health conditions and safeguard their right to return to work. People living with the double whammy of mental health conditions and pain are at a serious disadvantage in retaining employment security and contribute significantly to higher rates of absenteeism and presenteeism as compared to those without pain. On the other hand, there is irrefutable evidence to support the benefits of being in employment that addresses important psychosocial needs. The value, embedded in individual identity, social roles and status, is a critical component of the social gradients that impact mental health and associated mortality.

A positive development is the strategy’s position to eliminate stigma and discrimination by its affirmative support of vulnerable groups, and recognizing their specific or individualized needs to achieve social inclusion. However, this was a missed opportunity. The plan of action should have highlighted participation of people with lived experience, especially those in vulnerable situations, as equal stakeholders in assessing, reviewing and evaluating how the strategy shapes out in the future. Moreover, the plan mostly reaffirms on-going EU commitments to existing initiatives leaving member nations with individual adoption of strategies designed in their own capacities.

Societal Impact of Pain (SIP), a multi-stakeholder partnership between European Pain Federation EFIC and Pain Alliance Europe (PAE), published in 2023 a Joint Statement (Mental Health and Pain Policy, 2023) calling upon the European Commission and its member states to act and reduce SIP. Key recommendations include the following:

1. Introduce assessment of pain interference in people with mental health conditions such as major depression, anxiety, bipolar disorders, schizophrenia, psychosis and substance use disorders.
2. Provide integrated care to jointly address pain management and mental health services
3. Fund research on understanding the pain-mental health dynamic.
4. Enable early access to care interventions for high-risk people.
5. Train healthcare professionals and care givers on the mutually reinforcing relationship between pain and mental health conditions.
6. Encourage participation of people with lived experience in development of integrated care plans
7. Recognize positive impact of good work conditions & support re-integration of people into the workforce.
8. Develop policies that comprehensively address biological, psychological and social factors of pain in people living with the double burden.
9. Design campaigns for care providers and common public aimed at reducing the stigma and normalizing conversations around mental health and pain.
